# Squamocin modulates histone H3 phosphorylation levels and induces G_1 _phase arrest and apoptosis in cancer cells

**DOI:** 10.1186/1471-2407-11-58

**Published:** 2011-02-08

**Authors:** Chien-Chih Lee, Yi-Hsiung Lin, Wen-Hsin Chang, Pei-Chin Lin, Yang-Chang Wu, Jan-Gowth Chang

**Affiliations:** 1 Graduate Institute of Natural Products, College of Pharmacy, Kaohsiung Medical University, Kaohsiung, Taiwan; 2 Graduate Institute of Medicine, College of Medicine, Kaohsiung Medical University, Kaohsiung, Taiwan; 3 Department of Pediatrics, Kaohsiung Medical University Hospital, Kaohsiung, Taiwan; 4 Graduate Institute of Integrated Medicine, College of Chinese Medicine, China Medical University, Taichung, Taiwan; 5 Natural Medicinal Products Research Center, China Medical University Hospital, Taichung, Taiwan; 6 Institute of Clinical Medicine, College of Medicine, Kaohsiung Medical University, Kaohsiung, Taiwan; 7 Department of Laboratory Medicine, Kaohsiung Medical University Hospital, Kaohsiung, Taiwan; 8 Center of Excellence for Environmental Medicine, Kaohsiung Medical University, Kaohsiung, Taiwan; 9 Cancer Center, Kaohsiung Medical University Hospital, Kaohsiung, Taiwan

## Abstract

**Background:**

Histone modifications in tumorigenesis are increasingly recognized as important epigenetic factors leading to cancer. Increased phosphorylation levels of histone H3 as a result of aurora B and pMSK1 overexpression were observed in various tumors. We selected *aurora B *and *MSK1 *as representatives for testing various compounds and drugs, and found that squamocin, a bis-tetrahydrofuran annonaceous acetogenin, exerted a potent effect on histone H3 phosphorylation.

**Methods:**

GBM8401, Huh-7, and SW620 cells were incubated with 15, 30, and 60 μM squamocin for 24 h. The expressions of mRNA and proteins were analyzed by qRT-PCR and Western blotting, respectively. The cell viability was determined by an MTT assay. Cell cycle distribution and apoptotic cells were analyzed by flow cytometry.

**Results:**

Our results showed that squamocin inhibited the proliferation of GBM8401, Huh-7, and SW620 cells, arrested the cell cycle at the G_1 _phase, and activated both intrinsic and extrinsic pathways to apoptosis. In addition, we demonstrated that squamocin had the ability to modulate the phosphorylation levels of H3S10 (H3S10p) and H3S28 (H3S28p) in association with the downregulation of aurora B and pMSK1 expressions.

**Conclusions:**

This study is the first to show that squamocin affects epigenetic alterations by modulating histone H3 phosphorylation at S10 and S28, providing a novel view of the antitumor mechanism of squamocin.

## Background

Cancer is generally viewed as a set of diseases driven by genetic and epigenetic alterations. Epigenetics include the interrelated processes of DNA methylation, genomic imprinting, and histone modifications, and epigenetic aberrations may result in human cancers [1-4]. In the case of histone modifications, covalent modifications of the N-terminal tail domains, such as acetylation, methylation, and phosphorylation, are recognized as crucial epigenetic marks that modulate gene expression and genomic function. Aberrant histone modifications may be caused by improper activities of histone-modifying enzymes, leading to inappropriate expression of tumorigenesis-related genes [5,6].

In mammalian cells, phosphorylation of histone H3 is correlated with processes of chromosome condensation during mitosis and transcription. In addition, H3 phosphorylation occurs at two serine residues, S10 and S28, which can be mediated by histone kinases including mitogen- and stress-activated protein kinase 1 (MSK1) and aurora B kinase [7-9]. Recent studies demonstrated that phosphorylation of histone H3 at Ser10 (H3S10p) is critical during neoplastic transformation, and the steady state level of H3S10p is elevated in oncogene-transformed cells and human tumor cell lines [10-13]. Moreover, increased phosphorylation levels of H3S10 resulting from aurora B and pMSK1 overexpression is a precipitating factor in chromosome instability and may play a role in carcinogenesis [14,15]. It was suggested that regulating phosphorylation levels of histone H3 may be a possible target for cancer treatment.

**Figure 1:**
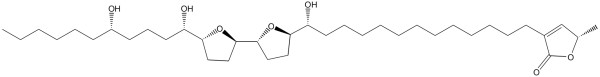
**Structure of squamocin**. Squamocin is characterized by a long alkyl chain bearing a terminal α, β-unsaturated γ-lactone ring, two tetrahydrofuran rings, and some oxygenated substitutes along the chain.

Under the assumption that targeting histone H3 phosphorylation by histone-modifying enzymes may have therapeutic potential for cancer treatment, we have been searching for small molecules that modulate enzymes involved in histone H3 phosphorylation in human cancer cells. Choosing *aurora B *and *MSK1 *as representatives to test various compounds and drugs, we found that squamocin (Figure 1) exerted a potent effect on histone H3 phosphorylation. We further used different cancer cell lines such as GBM8401, Huh-7, and SW620 to evaluate whether it has similar effects on different caners. We analyzed changes in the cell cycle and apoptosis, as well as histone H3 phosphorylation levels in association with expressions of these histone-modifying enzymes, in an effort to investigate the possible antitumor mechanism of squamocin.

## Methods

### Materials and Chemicals

Dulbecco's modified Eagle medium (DMEM), fetal bovine serum (FBS), trypan blue, penicillin G, and streptomycin were obtained from GIBCO BRL (Gaithersburg, MD, USA). 3-(4,5-Dimethylthiazol-2-yl)-2,5-diphenyltetrazolium bromide (MTT), dimethyl sulfoxide (DMSO), ribonuclease (RNase), and propidium iodide (PI) were purchased from Sigma-Aldrich (St. Louis, MO, USA). An Annexin V-FITC Staining Kit was purchased from Strong Biotech (Taipei, Taiwan). Antibodies against aurora B, H3S10p, and H3S28p were purchased from Abcam (Cambridge, UK). Antibodies against pERK, pMSK1, caspase-3, caspase-8, caspase-9, and GAPDH were obtained from Santa Cruz Biotechnology (Santa Cruz, CA, USA). Anti-PARP was purchased from Upstate Biotechnology (Charlottesville, VA, USA). Anti-mouse and anti-rabbit immunoglobulin G (IgG) peroxidase-conjugated secondary antibodies were purchased from Pierce (Rockford, IL, USA). Polyvinylidene difluoride (PVDF) membranes and an enhanced chemiluminescence (ECL) Western blotting detection kit were obtained from Amersham Life Science (Buckinghamshire, UK).

### Preparation of the squamocin solution

Squamocin was provided by Prof. Yang-Chang Wu, Graduate Institute of Natural Products, Kaohsiung Medical University, Kaohsiung, Taiwan. The structure of this compound was verified by means of mass spectrometry and spectroscopic techniques [16]. Squamocin was dissolved in DMSO (< 0.01%) and made up immediately prior to the experiments.

### Cell culture

The GBM8401, Huh-7, and SW620 cell lines were obtained from American Type Culture Collection (ATCC, Manassas, VA, USA), and are derived from brain, liver and colon cancers, respectively. Cells were maintained in DMEM which was supplemented with 10% FBS, 2 mM glutamine, and antibiotics (100 U/ml penicillin and 100 μg/ml streptomycin) at 37°C in a humidified atmosphere of 5% CO_2_.

### Cell growth inhibition assay

Cell viability was determined by an MTT assay, and results are presented as a percentage of the control. For the MTT assay, 10 μl of MTT (5 mg/ml) dye was directly added to the cell cultures. The medium was removed 2 h later, and cells were lysed with 100 μl of DMSO. The absorbance at 570 nm was read on a microplate reader.

### Flow cytometry

Externalization of phosphatidylserine (PS) and the membrane integrity were quantified using an Annexin V-FITC Staining Kit. Cells were washed twice with phosphate-buffered saline (PBS), and collected by centrifugation at 200 × *g *for 5 min at 25°C. Cells were resuspended in 100 μl of binding buffer and labeled with 2 μl of annexin V-FITC and PI for 15 min at 25°C. After labeling, cells were resuspended in 500 μl of binding buffer and detected on a flow cytometer using 488-nm excitation and a 515-nm band-pass filter for fluoresce detection and a filter > 600 nm for PI detection. To analyze the cell cycle distribution, cells were washed twice with PBS, collected by centrifugation at 200 × *g *for 5 min at 4°C, and fixed in 70% (v/v) ethanol at 4°C for 30 min. After fixation, cells were treated with 0.2 ml of the DNA extraction buffer (0.2 M Na_2_HPO_4 _and 0.1 M citric acid buffer; pH 7.8) for 30 min, centrifuged, and then resuspended in 1 ml of PI staining buffer (0.1% TritonX-100, 100 μg/ml RNase A, and 500 μg/ml PI in PBS) at 37°C for 30 min. Cells were detected using a flow cytometer and analyzed by FACScan and the Cell Quest program (Becton Dickinson, San Jose, CA, USA).

### Western blot analysis

Total proteins were extracted as previously described [17]. Proteins were extracted from the experimental and control samples and analyzed by sodium dodecylsulfate polyacrylamide gel electrophoresis (SDS-PAGE) as follows: after electrophoresis, proteins were transferred from the gel onto PVDF membranes. The membranes were blocked with a skim milk solution (5% skim milk in PBS) and agitated for 30 min at room temperature. Membranes were exposed to the primary antibody and agitated for 1 h at room temperature before being washed three times for 10-min periods with PBST (0.05% Tween 20 in PBS), and then incubated for 1 h with the secondary antibody at a 1:2500 dilution. After incubation with the antibody, the membranes were washed three times with PBST for 10 min each and then immersed in an ECL solution (combining solutions A and B of the ECL kit in a 1:1 ratio) with agitation for 1 min. After washing, the blots were developed by ECL.

### Quantitative real-time reverse-transcriptase polymerase chain reaction (qRT-PCR)

RNA was isolated from cultured cells, and the analysis was performed as previously described [18]. The PCR was performed in a final volume of 20 μl using a LightCycler instrument (Roche Diagnostics) according to the manufacturer's recommendations. The amounts of complementary (c)DNA were normalized to the housekeeping gene, *GAPDH*, to calculate the relative expressions of *aurora B *and *MSK1 *RNA (Table 1). Primers used to detect these genes were designed using ProbeFinder software http://www.roche.com and were synthesized by custom oligonucleotide synthesis (Genomics, Taipei, Taiwan). The qRT-PCR cycling parameters were set as follows: 40 cycles of 95°C for 10 s (denaturation), followed by 60°C for 30 s (annealing), and 72°C for 1 s (extension).

**Table 1:** Information about the primers and probes used in qRT-PCR

Gene	Forward primer	Reverse primer	Probe
*aurora B*	attgctgacttcggctggt	gtccagggtgccacacat	69
*GAPDH*	agccacatcgctcagacac	gcccaatacgaccaaatcc	60
*MSK1*	tggtgctgacagattttggt	caaaaggaatatgctctttcagtttc	5

### Statistical analysis

Results from multiple experiments are expressed as the mean ± standard error. The difference between the treatment and control groups was analyzed by Student's *t*-test. A probability (*p*) value of < 0.05 was considered significant.

## Results

### Squamocin decreased aurora B and pMSK1 RNA and protein expression levels

**Figure 2:**
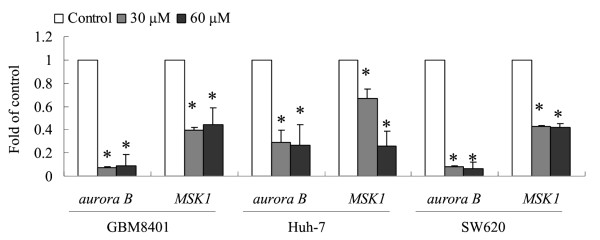
**Squamocin decreased expression levels of RNA of *aurora B *and *MSK1***. GBM841, Huh-7 and SW620 cells were incubated with 30 and 60 μM squamocin for 24 h. mRNA was extracted and detected by qRT-PCR. Data represent fold change versus controls, and values were normalized to *GAPDH*. Data are the mean of three independent experiments. * *p *< 0.05, compared to the control.

**Figure 3:**
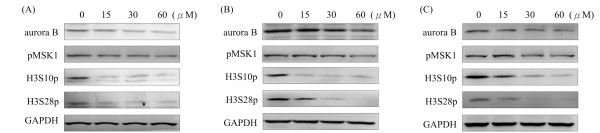
**Downregulation of aurora B, pMSK1, H3S10p, and H3S28p protein expression levels was observed with squamocin treatment**. Cells were incubated with 15, 30, and 60 μM squamocin for 24 h. Proteins were extracted and analyzed by Western blotting. GAPDH was used as a loading control. (A) GBM841 cells. (B) Huh-7 cells. (C) SW620 cells. Data are representative of three independent experiments.

Aurora B and MSK1 are thought to be involved in chromatin organization, gene expression, and carcinogenesis [14,15]. More than 20 compounds with cytotoxic effects were screened, and we found a compound, squamocin, isolated from several genera of the plant family Annonaceae, which decreased (m)RNA expression levels of *aurora B *and *MSK1 *in cancer cells. The expressions of *aurora B *and *MSK1 *were significantly downregulated in squamocin-treated GBM8401, Huh-7, and SW620 cells compared to the control (Figure 2). Similarly, squamocin treatment decreased the protein expression levels of aurora B and phosphorylated MSK1 (pMSK1) (Figure 3). These results imply that squamocin regulates aurora B and MSK1 activities at the transcriptional and translation levels.

### Squamocin downregulated phosphorylation levels of histone H3 at Ser10 and Ser28

In eukaryotes, aurora B and MSK1 are linked to the phosphorylation of H3S10 and H3S28 [7-9]. In order to investigate the effects of squamocin on H3S10p and H3S28p, cells were treated with squamocin for 24 h, and the protein expression levels were analyzed by Western blotting. The results showed that decreased H3S10p and H3S28p protein expression levels were detected in squamocin-treated cells (Figure 3). Our experiment revealed that squamocin treatment decreased phosphorylation of histone H3S10 and H3S28, as well as caused declines in the protein and RNA expression levels of aurora B and pMSK1. The modulation of H3S10 and H3S28 phosphorylation by aurora B and/or pMSK1 indicates that squamocin probably decreased the phosphorylation of H3S10 and H3S28 by downregulating aurora B and pMSK1 in cancer cells.

### Effects of squamocin on cell viability

**Figure 4:**
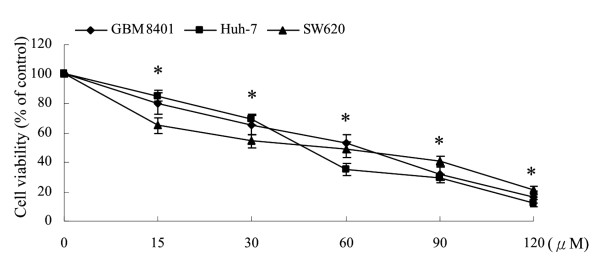
**Inhibition of cancer cell growth by squamocin**. GBM8401, Huh-7, and SW620 cells were treated with the indicated concentrations of squamocin, and cell viability was determined by an MTT assay. Data are the mean of three independent experiments. * *p *< 0.05, compared to the control.

The growth-inhibitory activity of squamocin was assessed by an MTT assay. GBM8401, Huh-7, and SW620 cells were treated with different concentrations (15~120 μM) of squamocin for 24 h. The results showed that squamocin-treated cancer cells exhibited significant loss of viability in dose-dependent manners (Figure 4). The 50% inhibitory concentrations (IC_50_) of GBM8401, Huh-7, and SW620 cells were 46.1, 39.4, and 40.4 μM, respectively.

### Squamocin arrested the cell cycle at the G_1 _phase and induced apoptosis

**Figure 5:**
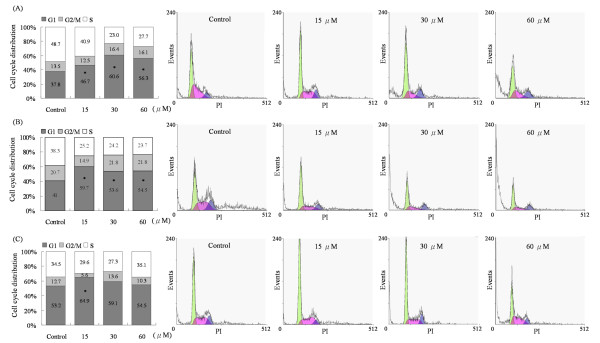
**Squamocin induced cell cycle arrest at the G_1 _phase**. Cells were treated with 15, 30, and 60 μM squamocin for 24 h, and then cells were stained with propidium iodide (PI) and analyzed for DNA content by flow cytometry. G_1_, S, and G_2_/M indicate cell phase. Cells without squamocin treatment served as a control. (A) GBM841 cells. (B) Huh-7 cells. (C) SW620 cells. Data are the mean of three independent experiments. * *p *< 0.05, compared to the control.

**Figure 6:**
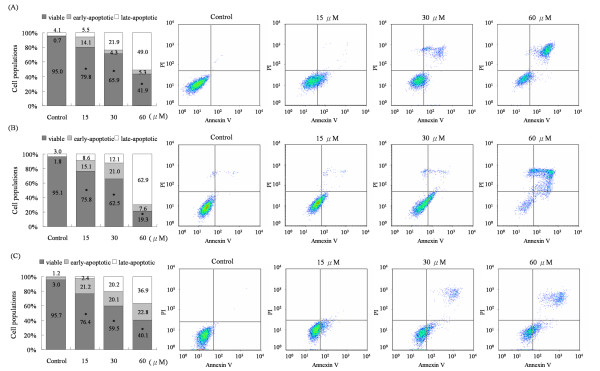
**High levels of early and late apoptosis were detected after squamocin treatment**. Cells were incubated with 15, 30, and 60 μM squamocin for 24 h. Apoptotic cells were determined by a PI and annexin V double-staining assay and analysis by flow cytometry. Annexin V^-^/PI^-^, annexin V^+^/PI^- ^and annexin V^+^/PI^+ ^cells were respectively considered to be viable, early-apoptotic, and late-apoptotic cells. Cells without squamocin treatment served as a control. (A) GBM841 cells. (B) Huh-7 cells. (C) SW620 cells. Data are the mean of three independent experiments. * *p *< 0.05, compared to the control.

**Figure 7:**
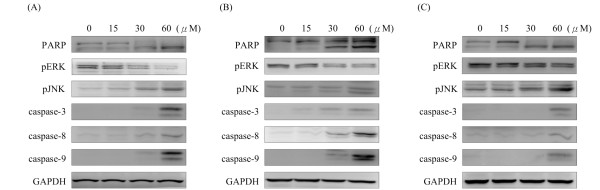
**Effects of squamocin on apoptosis**. Cells were treated with 15, 30, and 60 μM squamocin for 24 h. Proteins were extracted and analyzed by Western blotting. GAPDH was used as a loading control. Squamocin enhanced caspase-3, -8, and -9 activities, cleaved the functional protein of PARP, increased phosphorylation levels of ERK, and decreased phosphorylation levels of JNK. (A) GBM841 cells. (B) Huh-7 cells. (C) SW620 cells. Data are representative of three independent experiments.

To further evaluate the potential relevance of histone H3 phosphorylation in cancer therapy, we examined the effects of squamocin on cell growth and viability. Cells were treated with squamocin for 24 h, and the cell cycle distribution and apoptosis were measured by a flow cytometric analysis. Squamocin treatment significantly increased the population of G_1 _phase cells (Figure 5). Also, high levels of apoptosis were detected in squamocin-treated cells (Figure 6). As shown in Figure 5, treatment of cells with 0, 15, 30, and 60 μM of squamocin resulted in G_1 _phase accumulation of cells corresponding to 37.8%, 46.7%, 60.6% and 56.3%, respectively in GBM841 cells (Figure 5A), 41%, 59.7%, 53.6%, and 54.5%, respectively in Huh-7 cells (Figure 5B), and 53.2%, 64.9%, 59.1%, 54.5%, respectively in SW620 cells (Figure 5C). Moreover, squamocin-treated cells were stained with propidium iodide (PI) and annexin V to determine the apoptotic cells. Cells were differentiated among viable (annexin V, PI double negative), early-apoptotic (annexin V positive, PI negative) and late-apoptotic (annexin V, PI double positive) cells. Treatment of cells with 0, 15, 30, and 60 μM of squamocin increased the percentage of early apoptosis from 0.7% to 14.1%, 4.3%, and 5.3%, respectively and late apoptosis from 4.1% to 5.5%, 21.9%, and 49%, respectively in GBM8401 cells (Figure 6A), early apoptosis from 1.8% to 15.1%, 21%, and 7.6%, respectively and late apoptosis from 3.0% to 8.6%, 12.1%, and 62.9%, respectively in Huh-7 cells (Figure 6B), and early apoptosis from 3.0% to 21.2%, 20.1%, and 22.8%, respectively and late apoptosis from 1.2% to 2.4%, 20.2%, and 36.9%, respectively in SW620 cells (Figure 6C). Further, we extended our study to apoptosis-associated molecules and found that increasing levels of caspase-3, -8, and -9 activities and cleavage of poly ADP-ribose polymerase (PARP) were observed in squamocin-induced apoptosis (Figure 7). From the results, it is evident that squamocin affected cell cycle progression and apoptosis.

### Effects of squamocin on mitogen-activated protein kinase (MAPK)

The MAPK signaling pathway is implicated in a wide range of cellular functions, including cell proliferation, differentiation, survival, and apoptosis [19]. To assess whether activation of the MAPK signaling pathway is involved in squamocin-induced apoptosis, we investigated the activities of MAPK. The results showed that squamocin treatment significantly decreased ERK phosphorylation (pERK) levels and increased JNK phosphorylation (pJNK) levels (Figure 7). It was determined that activation of JNK affects members of the Bcl-2 family and activates caspases-3, -8, and -9 which results in apoptosis, whereas ERK is connected to cell survival [20,21] Our results indicate that inhibition of ERK and activation of JNK may participate in squamocin-induced apoptosis.

## Discussion

Annonaceous acetogenins are highly neurotoxic molecules and have been considered as new antitumor agents found in the plant family, the Annonaceae [22-24]. Extensive studies on annonaceous acetogenins indicated that these naturally occurring compounds possess a broad range of biological activities, including anticancer, antiparasitic, insecticidal, and immunosuppressive effects [25,26]. On the other hand, recent studies demonstrated that annonaceous acetogenins can be converted to activity-based probes for chemical proteomics. These probes were able to identify new putative targets including mitochondrial, cytosolic, and reticulum associated enzymes [27,28]. Squamocin, an annonaceous acetogenin, is a major component of various genera of the Annonaceae. Our previous studies showed that squamocin induces potent cytotoxicity against a variety of cancer cells [29]. In this report, we found that squamocin caused cell cycle arrest and apoptosis in three cancer cell lines. In addition, squamocin decreased the phosphorylation levels of H3S10 and H3S28 by downregulating aurora B and pMSK1 expressions, which might be the antitumor mechanism of squamocin.

Apoptosis, or programmed cell death, is known to participate in various biological processes. Two main apoptotic pathways were described: the mitochondrial (intrinsic) pathway and the death receptor (extrinsic) pathway. Both pathways induce activation of caspases and cause cell death. The intrinsic apoptotic pathway results from cytochrome *c *release from mitochondria into the cytosol and activates the initiator caspase-9 and the extrinsic apoptotic pathway results from activation of death-domain receptors and activates the initiator caspase-8 [30]. In addition, it is generally accepted that the biological activity of annonaceous acetogenins is the inhibition of nicotinamide adenine dinucleotide (NADH)-ubiquinone oxidoreductase (complex I) of the mitochondrial electron transport [25]. This inhibition suppresses mitochondrial membrane potential and ATP production as well as leads to intrinsic apoptotic pathway [31-33]. In our experiment, increasing levels of caspase-8 and -9 activities were detected in squamocin-treated cells, indicating that squamocin activated both intrinsic and extrinsic pathways to apoptosis in cancer cells.

In mammals, the ERK signaling pathway is the best studied of the MAPK pathways. Inappropriate regulation of the ERK pathway is connected to neoplastic transformation and tumor development. Most cancer-associated lesions that lead to constitutive ERK activation are associated with uncontrolled cell proliferation [34]. Thus, therapeutic targeting of individual components of the ERK pathway has attracted much attention for developing antitumor agents. Inhibition of ERK signaling could induce an early depletion in cellular ATP coincident with a loss of mitochondrial membrane potential, and lead to cytosolic release of mitochondrial proteins and caspases activation [35]. Besides, cell cycle arrest and apoptosis caused by ERK inhibition were observed in various cancer cell lines, indicating the potential utility in antitumor agent activity [36,37]. MSK1 is a serine/threonine protein kinase that can be phosphorylated by activated ERK (phosphorylated ERK) to promote kinase catalytic activity in response to multiple stimuli [38,39]. In our experiment, pERK downregulation was detected in squamocin-treated cells, and simultaneously caused a decline in the expression of pMSK1. It is probable that squamocin decreased the ERK cascade to reduce MSK1 phosphorylation.

Cancer cells frequently undergo mitosis, and many mitotic regulators are aberrantly expressed in these cells. Aurora B, a chromosomal segregation protein, is expressed during mitosis and carries out vital functions such as chromosome alignment, a spindle-checkpoint function, and cytokinesis [40]. Abnormally elevated expression of aurora B was detected in many human cancer cells, and this overexpression is linked to genomic instability which contributes to tumorigenesis [41]. Accordingly, aurora B inhibitors are important factors in cancer therapeutics. In this study, squamocin treatment decreased the expression of aurora B and also of pERK in cancer cells. The data suggest that squamocin may have potential therapeutic value in treating cancer.

Several studies demonstrated the roles of histone H3S10 and H3S28 phosphorylation in response to stimuli or other stresses [42,43]. In eukaryotes, histone H3 phosphorylation is altered along with cell mitosis. This phosphorylation is correlated with chromosome condensation prior to mitosis, and when chromosomes are dephosphorylated in mitosis, it induces chromosome decondensation [9]. In addition, it was reported that phosphorylation of H3S10 and H3S28 appears in the G_2_/M phase, and thus, both of them are widely used as cell cycle markers to index the G_2_/M stages [44,45]. Our experiment showed that histone H3 phosphorylation at S10 and S28 was reduced by squamocin, and the cell cycle was accordingly arrested at the G_1 _phase. This indicates that the decreased phosphorylation of H3S10 and H3S28 presumably caused a failure of cell cycle progression and resulted in G_1 _phase arrest with squamocin treatment.

It is well known that annonaceous acetogenins are the most potent inhibitors of the mitochondrial respiratory chain complex I [25]. The number of compounds that inhibit complex I is increasing, and parts of the diverse inhibitors, such as rotenoids, piericidins, and myxobacterial antibiotics could be gained from natural products. These inhibitors have been reported to display various activities in the inhibition of mitochondrial complex I [46]. Moreover, several reports have showed that the mitochondrial complex I inhibitor can reduce the phosphorylation levels of ERK [47], promote the activity of JNK [47,48] and caspases [49,50] as well as cause cell cycle arrest [51] and apoptosis [50]. Although the effects of these inhibitors were similar to the effects of squamocin, the squamocin treatment showed a new effect on histone modifications. Therefore, inhibition of mitochondrial complex I, modulation of histone or both may lead to the squamocin-induced cell cycle arrest and apoptosis, but the real mechanism needs further investigation.

## Conclusions

**Figure 8:**
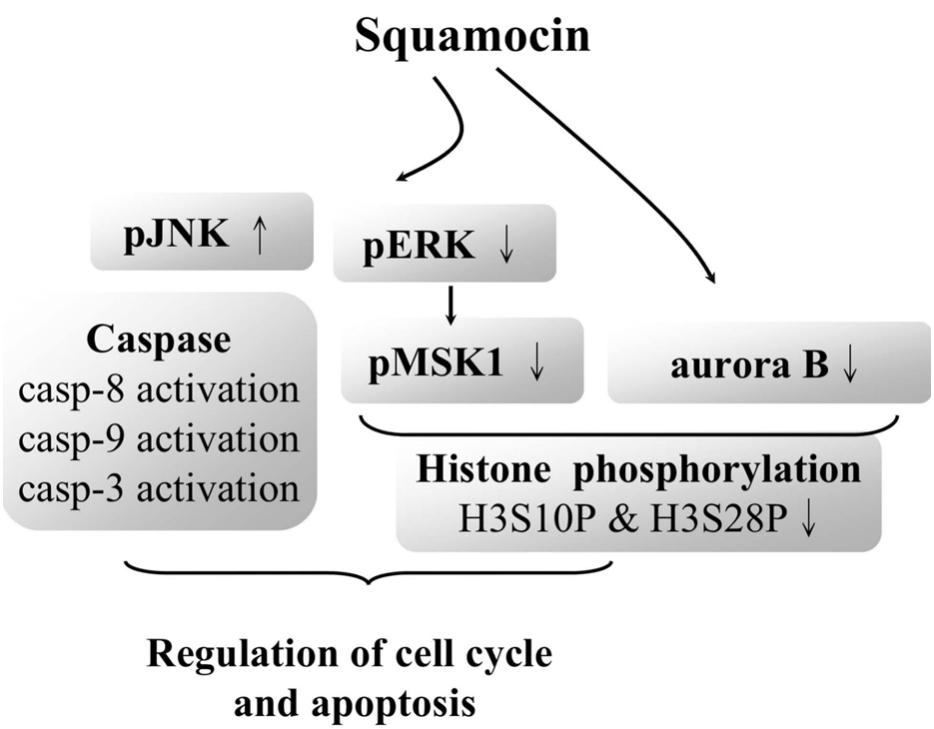
**Hypothetical schematic diagram of squamocin-induced cell cycle arrest and apoptosis in cancer cells**. Based on our results, squamocin could induce the activation of JNK and caspases, and decrease the phosphorylation levels of H3S10 and H3S28 by downregulating the expression of pERK, pMSK1, and aurora B. We proposed the hypothesis that histone dephosphorylation and activation of JNK and caspases contribute to squamocin induced cell cycle arrest and apoptosis.

Taken together, squamocin, a bis-tetrahydrofuran annonaceous acetogenin isolated from several genera of the plant family, the Annonaceae, induces G_1 _phase arrest and activates both intrinsic and extrinsic pathways to apoptosis in cancer cell lines. This study is the first to show that squamocin affects epigenetic alterations by modulating histone H3 phosphorylation at S10 and S28 (Figure 8), providing a novel view of the antitumor mechanism of squamocin.

## Competing interests

The authors declare that they have no competing interests.

## Authors' contributions

CCL performed the experiments and drafted the manuscript. YHL and WHC helped to design the study. PCL participated in the coordination of the study. YCW and JGC design the study. All authors read and approved the final manuscript.

## Pre-publication history

The pre-publication history for this paper can be accessed here:

http://www.biomedcentral.com/1471-2407/11/58/prepub

## References

[B1] Kouzarides T Chromatin modifications and their function Cell 2007 128 4 693 705 10.1016/j.cell.2007.02.005 17320507

[B2] Jones PA Baylin SB The fundamental role of epigenetic events in cancer Nat Rev Genet 2002 3 6 415 428 12042769 10.1038/nrg816

[B3] Jones PA Baylin SB The epigenomics of cancer Cell 2007 128 4 683 692 10.1016/j.cell.2007.01.029 17320506 PMC3894624

[B4] Hake SB Xiao A Allis CD Linking the epigenetic 'language' of covalent histone modifications to cancer Br J Cancer 2007 96 Suppl R31 39 17393583

[B5] Seligson DB Horvath S McBrian MA Mah V Yu H Tze S Wang Q Chia D Goodglick L Kurdistani SK Global levels of histone modifications predict prognosis in different cancers Am J Pathol 2009 174 5 1619 1628 10.2353/ajpath.2009.080874 19349354 PMC2671251

[B6] Strahl BD Allis CD The language of covalent histone modifications Nature 2000 403 6765 41 45 10.1038/47412 10638745

[B7] Perez-Cadahia B Drobic B Davie JR H3 phosphorylation: dual role in mitosis and interphase Biochem Cell Biol 2009 87 5 695 709 10.1139/O09-053 19898522

[B8] Peterson CL Laniel MA Histones and histone modifications Curr Biol 2004 14 14 R546 551 10.1016/j.cub.2004.07.007 15268870

[B9] Prigent C Dimitrov S Phosphorylation of serine 10 in histone H3, what for? J Cell Sci 2003 116 Pt 18 3677 3685 10.1242/jcs.00735 12917355

[B10] Graber MW Schweinfest CW Reed CE Papas TS Baron PL Isolation of differentially expressed genes in carcinoma of the esophagus Ann Surg Oncol 1996 3 2 192 197 10.1007/BF02305800 8646521

[B11] Chadee DN Hendzel MJ Tylipski CP Allis CD Bazett-Jones DP Wright JA Davie JR Increased Ser-10 phosphorylation of histone H3 in mitogen-stimulated and oncogene-transformed mouse fibroblasts J Biol Chem 1999 274 35 24914 24920 10.1074/jbc.274.35.24914 10455166

[B12] Choi HS Choi BY Cho YY Mizuno H Kang BS Bode AM Dong Z Phosphorylation of histone H3 at serine 10 is indispensable for neoplastic cell transformation Cancer Res 2005 65 13 5818 5827 10.1158/0008-5472.CAN-05-0197 15994958 PMC2227263

[B13] Kim HG Lee KW Cho YY Kang NJ Oh SM Bode AM Dong Z Mitogen- and stress-activated kinase 1-mediated histone H3 phosphorylation is crucial for cell transformation Cancer Res 2008 68 7 2538 2547 10.1158/0008-5472.CAN-07-6597 18381464 PMC2288657

[B14] Espino PS Pritchard S Heng HH Davie JR Genomic instability and histone H3 phosphorylation induction by the Ras-mitogen activated protein kinase pathway in pancreatic cancer cells Int J Cancer 2009 124 3 562 567 10.1002/ijc.23959 19004007

[B15] Adams RR Maiato H Earnshaw WC Carmena M Essential roles of Drosophila inner centromere protein (INCENP) and aurora B in histone H3 phosphorylation, metaphase chromosome alignment, kinetochore disjunction, and chromosome segregation J Cell Biol 2001 153 4 865 880 10.1083/jcb.153.4.865 11352945 PMC2192373

[B16] Chen CY Chang FR Chiu HF Wu MJ Wu YC Aromin-A, an Annonaceous acetogenin from Annona cherimola Phytochemistry 1999 51 3 429 433 10.1016/S0031-9422(99)00002-3 10382318

[B17] Chan CH Ko CC Chang JG Chen SF Wu MS Lin JT Chow LP Subcellular and functional proteomic analysis of the cellular responses induced by Helicobacter pylori Mol Cell Proteomics 2006 5 4 702 713 16401634 10.1074/mcp.M500029-MCP200

[B18] Andreassi C Angelozzi C Tiziano FD Vitali T De Vincenzi E Boninsegna A Villanova M Bertini E Pini A Neri G Phenylbutyrate increases SMN expression in vitro: relevance for treatment of spinal muscular atrophy Eur J Hum Genet 2004 12 1 59 65 10.1038/sj.ejhg.5201102 14560316

[B19] Kim EK Choi EJ Pathological roles of MAPK signaling pathways in human diseases Biochim Biophys Acta 2010 1802 4 396 405 20079433 10.1016/j.bbadis.2009.12.009

[B20] Junttila MR Li SP Westermarck J Phosphatase-mediated crosstalk between MAPK signaling pathways in the regulation of cell survival FASEB J 2008 22 4 954 965 10.1096/fj.06-7859rev 18039929

[B21] Liu CJ Lo JF Kuo CH Chu CH Chen LM Tsai FJ Tsai CH Tzang BS Kuo WW Huang CY Akt mediates 17beta-estradiol and/or estrogen receptor-alpha inhibition of LPS-induced tumor necresis factor-alpha expression and myocardial cell apoptosis by suppressing the JNK1/2-NFkappaB pathway J Cell Mol Med 2009 13 9B 3655 3667 10.1111/j.1582-4934.2009.00669.x 20196785 PMC4516514

[B22] Caparros-Lefebvre D Steele J Kotake Y Ohta S Geographic isolates of atypical Parkinsonism and tauopathy in the tropics: possible synergy of neurotoxins Mov Disord 2006 21 10 1769 1771 10.1002/mds.21024 16874753

[B23] Kotake Y Okuda K Kamizono M Matsumoto N Tanahashi T Hara H Caparros-Lefebvre D Ohta S Detection and determination of reticuline and N-methylcoculaurine in the Annonaceae family using liquid chromatography-tandem mass spectrometry J Chromatogr B Analyt Technol Biomed Life Sci 2004 806 1 75 78 10.1016/j.jchromb.2004.03.017 15149614

[B24] Alali FQ Liu XX McLaughlin JL Annonaceous acetogenins: recent progress J Nat Prod 1999 62 3 504 540 10.1021/np980406d 10096871

[B25] Bermejo A Figadere B Zafra-Polo MC Barrachina I Estornell E Cortes D Acetogenins from Annonaceae: recent progress in isolation, synthesis and mechanisms of action Nat Prod Rep 2005 22 2 269 303 10.1039/b500186m 15806200

[B26] Kojima N Tanaka T Medicinal chemistry of Annonaceous acetogenins: design, synthesis, and biological evaluation of novel analogues Molecules 2009 14 9 3621 3661 10.3390/molecules14093621 19783948 PMC6254973

[B27] Derbre S Roue G Poupon E Susin SA Hocquemiller R Annonaceous acetogenins: the hydroxyl groups and THF rings are crucial structural elements for targeting the mitochondria, demonstration with the synthesis of fluorescent squamocin analogues Chembiochem 2005 6 6 979 982 10.1002/cbic.200400396 15861433

[B28] Derbre S Gil S Taverna M Boursier C Nicolas V Demey-Thomas E Vinh J Susin SA Hocquemiller R Poupon E Highly cytotoxic and neurotoxic acetogenins of the Annonaceae: new putative biological targets of squamocin detected by activity-based protein profiling Bioorg Med Chem Lett 2008 18 21 5741 5744 10.1016/j.bmcl.2008.09.091 18851912

[B29] Liaw CC Wu TY Chang FR Wu YC Historic Perspectives on Annonaceous Acetogenins from the Chemical Bench to Preclinical Trials Planta Med 2010 76 13 1390 404 10.1055/s-0030-1250006 20577943

[B30] Philchenkov A Caspases: potential targets for regulating cell death J Cell Mol Med 2004 8 4 432 444 10.1111/j.1582-4934.2004.tb00468.x 15601572 PMC6740296

[B31] Duval RA Poupon E Romero V Peris E Lewin G Cortes D Brandt U Hocquemiller R Analogues of cytotoxic squamocin using reliable reactions: new insights into the reactivity and role of the α,β-unsaturated lactone of the annonaceous acetogenins Tetrahedron 2006 62 26 6258 6257 10.1016/j.tet.2006.04.066

[B32] Duval RA Poupon E Brandt U Hocquemiller R Remarkable substituent effect: beta-aminosquamocin, a potent dual inhibitor of mitochondrial complexes I and III Biochim Biophys Acta 2005 1709 3 191 194 10.1016/j.bbabio.2005.07.011 16139789

[B33] Derbre S Duval R Roue G Garofano A Poupon E Brandt U Susin SA Hocquemiller R Semisynthesis and screening of a small library of pro-apoptotic squamocin analogues: selection and study of a benzoquinone hybrid with an improved biological profile ChemMedChem 2006 1 1 118 129 10.1002/cmdc.200500011 16892343

[B34] Dhillon AS Hagan S Rath O Kolch W MAP kinase signalling pathways in cancer Oncogene 2007 26 22 3279 3290 10.1038/sj.onc.1210421 17496922

[B35] Monick MM Powers LS Barrett CW Hinde S Ashare A Groskreutz DJ Nyunoya T Coleman M Spitz DR Hunninghake GW Constitutive ERK MAPK activity regulates macrophage ATP production and mitochondrial integrity J Immunol 2008 180 11 7485 7496 18490749 10.4049/jimmunol.180.11.7485 PMC2410094

[B36] Roy SK Srivastava RK Shankar S Inhibition of PI3K/AKT and MAPK/ERK pathways causes activation of FOXO transcription factor, leading to cell cycle arrest and apoptosis in pancreatic cancer J Mol Signal 2010 5 10 10.1186/1750-2187-5-10 20642839 PMC2915986

[B37] Nishioka C Ikezoe T Yang J Yokoyama A Inhibition of MEK signaling enhances the ability of cytarabine to induce growth arrest and apoptosis of acute myelogenous leukemia cells Apoptosis 2009 14 9 1108 1120 10.1007/s10495-009-0372-4 19548087

[B38] Thomson S Clayton AL Hazzalin CA Rose S Barratt MJ Mahadevan LC The nucleosomal response associated with immediate-early gene induction is mediated via alternative MAP kinase cascades: MSK1 as a potential histone H3/HMG-14 kinase EMBO J 1999 18 17 4779 4793 10.1093/emboj/18.17.4779 10469656 PMC1171550

[B39] Dyson MH Thomson S Inagaki M Goto H Arthur SJ Nightingale K Iborra FJ Mahadevan LC MAP kinase-mediated phosphorylation of distinct pools of histone H3 at S10 or S28 via mitogen- and stress-activated kinase 1/2 J Cell Sci 2005 118 Pt 10 2247 2259 10.1242/jcs.02373 15870105

[B40] Yeung SC Gully C Lee MH Aurora-B kinase inhibitors for cancer chemotherapy Mini Rev Med Chem 2008 8 14 1514 1525 10.2174/138955708786786480 19075809

[B41] Katayama H Brinkley WR Sen S The Aurora kinases: role in cell transformation and tumorigenesis Cancer Metastasis Rev 2003 22 4 451 464 10.1023/A:1023789416385 12884918

[B42] Mahadevan LC Willis AC Barratt MJ Rapid histone H3 phosphorylation in response to growth factors, phorbol esters, okadaic acid, and protein synthesis inhibitors Cell 1991 65 5 775 783 10.1016/0092-8674(91)90385-C 2040014

[B43] Lee YJ Shukla SD Histone H3 phosphorylation at serine 10 and serine 28 is mediated by p38 MAPK in rat hepatocytes exposed to ethanol and acetaldehyde Eur J Pharmacol 2007 573 1-3 29 38 10.1016/j.ejphar.2007.06.049 17643407 PMC2723821

[B44] Juan G Traganos F James WM Ray JM Roberge M Sauve DM Anderson H Darzynkiewicz Z Histone H3 phosphorylation and expression of cyclins A and B1 measured in individual cells during their progression through G2 and mitosis Cytometry 1998 32 2 71 77 10.1002/(SICI)1097-0320(19980601)32:2<71::AID-CYTO1>3.0.CO;2-H 9627219

[B45] Goto H Yasui Y Nigg EA Inagaki M Aurora-B phosphorylates Histone H3 at serine28 with regard to the mitotic chromosome condensation Genes Cells 2002 7 1 11 17 10.1046/j.1356-9597.2001.00498.x 11856369

[B46] Degli Esposti M Inhibitors of NADH-ubiquinone reductase: an overview Biochim Biophys Acta 1998 1364 2 222 235 10.1016/S0005-2728(98)00029-2 9593904

[B47] Kwak HB Lee BK Oh J Yeon JT Choi SW Cho HJ Lee MS Kim JJ Bae JM Kim SH Inhibition of osteoclast differentiation and bone resorption by rotenone, through down-regulation of RANKL-induced c-Fos and NFATc1 expression Bone 2010 46 3 724 731 10.1016/j.bone.2009.10.042 19900598

[B48] Deng YT Huang HC Lin JK Rotenone induces apoptosis in MCF-7 human breast cancer cell-mediated ROS through JNK and p38 signaling Mol Carcinog 2010 49 2 141 151 19777565 10.1002/mc.20583

[B49] Hoglinger GU Lannuzel A Khondiker ME Michel PP Duyckaerts C Feger J Champy P Prigent A Medja F Lombes A The mitochondrial complex I inhibitor rotenone triggers a cerebral tauopathy J Neurochem 2005 95 4 930 939 10.1111/j.1471-4159.2005.03493.x 16219024

[B50] Lyamzaev KG Izyumov DS Avetisyan AV Yang F Pletjushkina OY Chernyak BV Inhibition of mitochondrial bioenergetics: the effects on structure of mitochondria in the cell and on apoptosis Acta Biochim Pol 2004 51 2 553 562 15218549

[B51] Bai J Nakamura H Ueda S Kwon YW Tanaka T Ban S Yodoi J Proteasome-dependent degradation of cyclin D1 in 1-methyl-4-phenylpyridinium ion (MPP+)-induced cell cycle arrest J Biol Chem 2004 279 37 38710 38714 10.1074/jbc.M403329200 15247282

